# Editorial: Signaling pathways and brain circuitry underlying circadian rhythms and sleep

**DOI:** 10.3389/fnins.2025.1558246

**Published:** 2025-02-07

**Authors:** Sydney Aten, Mino D. C. Belle, Cecilia G. Diniz Behn

**Affiliations:** ^1^Department of Neurology, Beth Israel Deaconess Medical Center, Harvard Medical School, Boston, MA, United States; ^2^Center for Biological Timing, Division of Neuroscience, School of Biological Sciences, Faculty of Biology, Medicine and Health, The University of Manchester, Manchester, United Kingdom; ^3^Department of Applied Mathematics and Statistics, Colorado School of Mines, Golden, CO, United States; ^4^Department of Pediatrics, University of Colorado Anschutz Medical Campus, Aurora, CO, United States

**Keywords:** circadian rhythm, sleep, mathematical modeling, calcium, rapid eye movement (REM) sleep, non-rapid eye movement (NREM) sleep, molecular/cellular

## Introduction

Circadian rhythms occur on a 24-h cycle and modulate physiological and behavioral processes, such as the sleep/wake cycle, across the day. The focus of this Research Topic is to highlight recent findings on the circuits and pathways that underlie or modulate circadian rhythms and sleep, including genetic, molecular/cellular, electrophysiological, neuroanatomical, pharmacological, and mathematical approaches. The Research Topic includes six papers that focus on three themes: (1) circadian modulation at different spatial scales (Hiro et al.; Aten et al.); (2) genetics of sleep/wake cycles (Kobayashi et al.; Doldur-Balli et al.); and (3) characterization and regulation of sleep stages (Yun et al.; Ginsberg et al.). Below, we provide an overview of each paper.

## Circadian rhythms: from signaling molecules to sexual behavior

Circadian rhythms are driven by a transcriptional/translational feedback loop (TTFL) composed of core clock genes and their products that manifest across many spatial/temporal scales from molecular to complex animal behavioral rhythms. At the molecular scale, Hiro et al. investigated circadian rhythm coordination in the suprachiasmatic nucleus (SCN), the location of the mammalian master circadian clock. SCN neurons exhibit circadian rhythms in cytosolic Ca^2+^, serving as a mediator in signaling pathways linking SCN electrical activity to TTFL gene expression (Colwell, 2011; Welsh et al., 2010; Mohawk et al., 2012; Takahashi, 2017). However, Ca^2+^ modulatory mechanisms between the cytosol and nucleus remain obscure. To better understand the mechanistic underpinnings of nuclear Ca^2+^ regulation, Hiro et al. performed dual-color nuclear and cytosolic Ca^2+^ imaging of mouse SCN neurons, using genetically encoded Ca^2+^ sensors (Chen et al., 2013; Dana et al., 2016). Results revealed a strong nuclear Ca^2+^ rhythm which was in-phase with the cytosolic Ca^2+^ rhythm, both within single neurons and SCN-wide subregions. Their results also suggest that circadian Ca^2+^ rhythms are driven from the extracellular space, although more work is needed to understand the mechanisms involved.

At the behavioral level, Aten et al., review time-of-day dependence in sexual and reproductive behaviors and connections to circadian rhythms in reproductive hormones in rodents, non-human primates, and humans. They also propose a novel neural circuit—spanning the SCN and sub-paraventricular zone to the ventromedial hypothalamus and medial preoptic area—that may control sexual behaviors across the circadian day. Lastly, they highlight studies that examine time-of-day differences in human sexual behaviors and discuss how circadian dysfunction negatively impacts reproduction.

## Genes that modulate the sleep/wake cycle

Sleep is an evolutionarily conserved behavioral state (Joiner, 2016), and model organisms like *Drosophila* and zebrafish have been widely used to genetically dissect sleep (Cirelli and Bushey, 2008; Zhdanova, 2006). Motivated by the finding that a mutation (*Sleepy*) in the *salt-inducible kinase 3 (Sik3)* gene was previously shown to alter sleep in mice (Funato et al., 2016), Kobayashi et al. tested whether a phosphorylation-deficient mutant form of the *Sik3* gene (“*Sik3-SA*”) might also affect sleep regulation in flies. They found that overexpression of this *Sik3-SA* mutation in all *Drosophila* neurons increased sleep while *o*verexpression of *Sik3-SA* specifically in pigment-dispersing factor (PDF), which modulates circadian rhythms in *Drosophila* (Guo et al., 2016), increased daytime sleep and decreased the circadian rhythm amplitude. Their results also suggest that *Sik3-SA* alters sleep regulation by PDF neurons in *Drosophila*, but future experiments are necessary to understand the relationship between *Sik3-SA* and PDF neuron functionality.

Sleep regulation may also be affected by hyperpolarization-activated cyclic nucleotide-gated (HCN) channels. In zebrafish, Doldur-Balli et al. investigated HCN “pacemaker” channels (Wobig et al., 2020), which are also known to modulate sleep/wake cycles (Lewis and Chetkovich, 2011; Sartiani et al., 2017). The authors tested the effects of three HCN channel inhibitors, Ivabradine, Zatebradine Hydrochloride, and ZD7288, on sleep/wake cycles in zebrafish and found that Ivabradine (at 0.1 μM) led to a shorter latency to daytime sleep relative to vehicle treatment. In addition, Zatebradine Hydrochloride (at 30 μM) led to a decrease in average daytime activity, and ZD7288 (at 4.5 μM) led to a nighttime sleep increase. While these three compounds are used to reduce heart rate (Novella Romanelli et al., 2016), this is the first study to report the effects of HCN channel inhibition in zebrafish—results that could provide insight into potential therapeutics that may impact sleep function.

## Electrophysiological and mathematical approaches to measure and predict sleep

Previous studies of local field potential (LFP) frequency band power and single-unit dynamics have helped advance our knowledge of sleep/wake stages, including rapid eye movement (REM) sleep and non-REM (NREM) sleep, and other complex brain functions (Brown et al., 2012; Rasch and Born, 2013). Yun et al. contribute to this literature by examining power spectral density of LFPs in the primary motor cortex in *Macaca nemestrina* monkeys. Single-unit activity was tracked simultaneously with six different frequency bands, and behavioral state-dependent changes in cross-frequency coupling were determined. They showed that LFP bands depend on the macaque's behavioral state, providing a foundation for future work that examines the function of various LFP bands in wake, NREM sleep, and REM sleep.

Although it is known that mammals alternate between REM and NREM sleep, the mechanisms driving this alternation have not been established (Le Bon, 2020). One hypothesis posits that a homeostatic drive for REM sleep increases during NREM sleep and dissipates during REM sleep (Benington et al., 1994; Heller, 2021). Ginsberg et al. build on previous work from Park et al. (2021) to define a new measure of REM sleep propensity. Their analyses of spontaneous mouse sleep data suggests that time spent in NREM sleep may increase the propensity to transition into REM sleep in a homeostatic “hourglass-like” manner, but only for a limited range of NREM sleep durations. Future work is needed to validate this REM propensity measure in other species or contexts such as sleep deprivation.

## Conclusion

Insights from the six papers in this Research Topic should provide direction for future experiments that will translate these fundamental-research-based methods into tools that could be used clinically to improve sleep and circadian-related health outcomes in humans.
